# ARHGEF3 regulates the stability of ACLY to promote the proliferation of lung cancer

**DOI:** 10.1038/s41419-022-05297-4

**Published:** 2022-10-14

**Authors:** Feifei Zhou, Wenqian Ai, Yixing Zhang, Qifan Hu, Mingxi Gan, Jian-Bin Wang, Tianyu Han

**Affiliations:** 1 Jiangxi Institute of Respiratory Disease, The First Affiliated Hospital of Nanchang University, 330006 Nanchang, P. R. China; 2 School of Basic Medical Sciences, Nanchang University, 330031 Nanchang, P. R. China; 3 Jiangxi Clinical Research Center for Respiratory Diseases, 330006 Nanchang, P. R. China; 4 Jiangxi Hospital of China-Japan Friendship Hospital, 330052 Nanchang, P. R. China

**Keywords:** Non-small-cell lung cancer, Acetylation

## Abstract

Rho GTPases play an essential role in many cellular processes, including cell cycle progress, cell motility, invasion, migration, and transformation. Several studies indicated that the dysregulation of Rho GTPase signaling is closely related to tumorigenesis. Rho GEFs considered being positive regulators of Rho GTPase, promoting the dissociation of Rho protein from GDP and binding to GTP, thus activating the downstream signaling pathway. Herein, we demonstrated that ARHGEF3, a member of the Rho GEFs family, played an important role in non-small cell lung cancer (NSCLC). We found that ARHGEF3 was highly expressed in non-small cell lung cancer and facilitated cancer cell proliferation of NSCLC cells in vitro and in vivo. Further studies demonstrated that ARHGEF3 enhanced the protein homeostasis of ATP-citrate lyase (ACLY) by reducing its acetylation on Lys17 and Lys86, leading to the dissociation between ACLY and its E3 ligase-NEDD4. Interestingly, this function of ARHGEF3 on the protein homeostasis of ACLY was independent of its GEF activity. Taken together, our findings uncover a novel function of ARHGEF3, suggesting that ARHGEF3 is a promising therapeutic target in non-small cell lung cancer.

## Introduction

Studies have shown that Rho protein regulates a variety of cellular processes, and abnormal activation of Rho GTPase is associated with oncogenesis and metastasis [1]. Classical Rho GTPases act as molecular switches, cycling between the activation state bound to GTP and the inactivation state bound to GDP. The activation of Rho GTPases is regulated by guanine exchange factors (GEFs). ARHGEF3, also called XPLN, is a guanine nucleotide exchange factor found in platelets, leukemic and neuronal tissues. It is a protein composed of DH and PH domains and contains 526 amino acids. The DH domain with N-terminal is mainly responsible for binding to Rho GTPases and has Rho GEFs catalytic activity, while the PH domain with C-terminal plays a major role in the subcellular localization of GEFs [2]. Arthur WT et al. reported that ARHGEF3 selectively activated RhoA and RhoB in vitro [3]. ARHGEF3 was an endogenous inhibitory molecule of mTORC2 and regulated cell survival and differentiation of skeletal muscle cells via inhibiting phosphorylation of AKT [4]. Moreover, ARHGEF3 played a key role in skeletal muscle regeneration through autophagy [5]. In terms of tumors, ARHGEF3 controlled HDACi-induced differentiation of lymphoma cells through RhoA/ROCK/JNK signaling pathway in acute myeloid leukemias [6]. ARHGEF3 accelerated the progression of nasopharyngeal carcinoma by promoting the expression of anti-apoptotic protein BIRC and inhibiting the expression of apoptotic protein caspase 3 [7]. However, the function of ARHGEF3 in lung cancer has not been reported, and the effects of ARHGEF3 on cancer metabolism remain unclear.

ATP-citrate lyase (ACLY), a cytoplasmic enzyme, converts citrate into acetyl-CoA and oxaloacetic acid (OAA). Acetyl-CoA is required for lipogenesis and cholesterogenesis. Besides, acetyl-CoA is involved in the acetylation of proteins. Thus, ACLY is a key enzyme in cancer metabolism [8]. Previous studies have proved that the expressions of ACLY were increased in colorectal cancer, bladder cancer, lung cancer, glioblastoma, etc., and promoted tumor growth [9–12]. Elucidating the regulation mechanisms of ACLY in cancer helps people to further understand its function in cancer initiation and progression. In this study, we found that ARHGEF3 was a new regulator of ACLY in non-small cell lung cancer, which stabilized ACLY by reducing its acetylation and decreasing its ubiquitination, thus promoting tumor growth and progression. In conclusion, our study suggests that ARHGEF3 is a promising therapeutic target for NSCLC.

## Materials and methods

### Cell culture and transfection

Human lung epithelial cell line BEAS-2B, NSCLC cell lines (H1299/A549/PC9/H292), and HEK293T cells were purchased from the American Type Culture Collection (ATCC, Manassas, VA, USA). DMEM medium (C11995500BT; Gibco) was used to culture HEK293T cells. RPMI 1640 medium (C11875500BT; Invitrogen) was used to culture BEAS-2B, H1299, A549, PC9, and H292 cells. The cells were fed in a medium containing 10% fetal bovine serum (10091148; Gibco) and placed in an incubator containing 5% CO_2_ at 37 °C. The cells were cultured in six-well plates until the density approximately reached 70–80%. Then, the plasmids or siRNAs were transfected into cells according to the instructions of the transfection reagent kit (2102-100/2103-100; Pufei).

### Plasmid construct and RNAi

The human full-length fragment of ARHGEF3 (NM_001128615.2) and mutants (L269E, W440L) was subcloned into the mammalian expression vector pCMV-N-HA to obtain the expression plasmid pCMV-N-HA-ARHGEF3, pCMV-N-HA-ARHGEF3-L269E, and pCMV-N-HA-ARHGEF3-W440L mutants and examined by sequencing. The pCMV-HA-SIRT2 plasmid was purchased from Miaoling Plasmid Sharing Platform. The PRK7-N-Flag-ACLY and mutants (K17R, K86R, K540R/546 R/554 R, K948R/962 R, K968R/978 R, K1077R) of ACLY were ordered from Tsingke Biotechnology Co., Ltd. The siRNAs targeting ARHGEF3 (HSS121363, HSS121365, HSS181740) were purchased from Thermo Fisher (17537881). The ARHGEF3 human shRNA plasmid was purchased from Origene (TL306606). ACLY siRNA was purchased from Origene (SR300035).

### Patients and clinical tissue samples

Fresh NSCLC tissue and para-cancer tissue samples were obtained from the Department of Thoracic Surgery at the First Affiliated Hospital of Nanchang University. Fresh tissue samples were immediately placed in liquid nitrogen and cryopreserved at −80 °C for subsequent experimental analysis. All experiments with human tissue samples were approved by the Ethics Committee of the First Affiliated Hospital of Nanchang University (Nanchang, China).

### Western blot and antibodies

Total protein extracts according to our previous study [13]. The primary antibodies used are as follows: ARHGEF3 (PA598942; Thermofisher), ACLY (67166-1-lg; Proteintech), HA-tag (26183; Thermofisher), Flag-tag (20543-1-AP; Proteintech), β-Actin (TA811000; Origene), ubiquitin (10201-2-AP; Proteintech), acetylation (06-933; Millipore), and GAPDH (TA802519; Origene).

### Quantitative real-time PCR (qPCR)

Total RNA was extracted from fresh cells using TRIzol reagent (15596018; Invitrogene). Reverse transcription was performed using the M5 Super PLUS qPCR RT kit (gDNA remover) (MF166-PLUS; Origene) according to the manufacturer’s instructions. RT-qPCR was performed using ChamQ Universal SYBR qPCR Master Mix kit (Q711-02; Vazyme) and analyzed using GraphpadPrism software. The target gene mRNA expression level was normalized to GAPDH mRNA expression level, and was quantified by $\Delta \Delta$CT method.

### Immunohistochemistry (IHC)

The sample was fixed, dehydrated, and embedded in wax. The tissue of the mouse xenograft specimen was cut into 3 μm slices and mounted on slides for histopathological evaluation. Briefly, tissue was dewaxed, hydrated, and dripped with 3% hydrogen peroxide. Then, EDTA buffer was used to treat tissue and exposed to high temperature for 30 minutes. Applying the primary antibodies to the tissue in accordance with the standard antigen retrieval protocol. The primary antibody Ki67 (ab15580; Abcam) was incubated overnight at 4 °C. PBS was washed three times, and the secondary antibody was incubated for 1 h. Chromogenic agents were added to the slices and developed for observation. ImageScope software was used to analyze tissue microarray images.

### Immunoprecipitation

NP-40 lysate (56741-50ML-F; Sigma) was used to lysate cells and extract proteins. Protein G agarose (11243233001; Roche) was added to the protein solution to pre-purify the protein for 30 min, centrifuging and incubating the protein solution with protein G agarose beads and indicated antibodies at 4 °C overnight. Next, the immune complexes bound to the microbeads were cleaned with lysis buffer, and a western blot was performed.

### Cell growth assay

Cell growth experiments were performed according to our previous study [13].

### Clone formation assay

Five hundred cells were inoculated in six-well plates. After forming a certain number of single visible clones, the cells were fixed with 4% paraformaldehyde for 15 min and stained with 0.1% crystal violet for 5 s. Then the background was cleaned with PBS, pictures were taken, and Image J was used for counting analysis.

### Flow cytometry analysis

The cells were collected, washed with PBS, and fixed in 70% ethanol at −20 °C for 2 h, the cell suspension was centrifuged at 850 rpm for 3 min. Then, adding propidium iodide (PI) (P4864; Sigma), and incubating for 15 min at 37 °C away from light. Finally, the samples were analyzed using BD flow cytometry (Becton Dickinson).

### Nuclear and cytosol extraction

The Nuclear and Cytoplasmic Extract Kit (P0028; Beyotime) was used for the extraction of cytoplasmic and nuclear proteins. The cells were scraped off with a cell scraper, centrifuged at 800 rpm at 4 °C, and the cell precipitate was retained. 200 μl of cytoplasmic protein extraction reagent A with PMSF (WB0181; Dingguo) was added to 20 μl of cell precipitate and vortexed violently to make the cell precipitate completely suspended and dispersed. After 10–15 min in the ice bath, 10 μl cytoplasmic protein extraction reagent B was added, and the reagent was vortexed at the highest speed, then 1000–16,000 × *g* was centrifuged at 4 °C. The supernatant was cytoplasmic protein. A nuclear protein extraction reagent containing PMSF was added to the cell precipitate, and the cells were vortexed violently at high speed every 1–2 min for 15–30 s for a total of 30 min. Centrifugation at 12,000–16,000 × *g* for 10 min at 4 °C, and the supernatant was nuclear protein.

### Immunofluorescence assay

Cells were spread into 24-well-plate with a cell slide and grew for 18–24 h. The cell slides were washed with 1× PBS for 5 min each time, then 4% paraformaldehyde was added and fixed at room temperature for 20 min. 0.2% Triton-X 100 was added and drilled for 20 min at room temperature, the cells were washed three times with PBS. Sealing with 5% BSA at room temperature for 30 min. The blocking fluid was removed, and the primary antibody was incubated overnight. The primary antibody was discarded, and the cells were washed with PBS, three times in 5 min, then the corresponding fluorescent secondary antibodies were incubated at room temperature for 1 h. The cells were washed three times with PBS, and then DAPI (0100-20; Southern Biotech) was added to the cells for staining. Finally, the confocal microscope was used to photograph.

### Mass spectrometry analysis

H1299 cells were transfected with Vector and ARHGEF3 plasmids, respectively. When the cell number reached 5 × 10^6^, proteins were collected as described in the immunoprecipitation assay. Then the protein samples were sent to Novogene for mass spectrometry analysis.

### Measurement of Acetyl-CoA content

The cells were lysed with NP-40 buffer containing PMSF (WB0181; Dingguo), then the cell suspension was ultrasonicated on ice for four times, 5 s each time. Centrifugation 12,000 × *g* at 4 °C for 10 min. The supernatant was determined by Acetyl-Coenzyme A assay kit (MAK039; Sigma).

### Fatty acid content determination

Using NP-40 buffer with PMSF to lysis cells. Cell supernatant was used for ELSA assay. Based on the human FFA kit (H02909; Lanpai Biotechnology) to detect the content of free fatty acid.

### Subcutaneous xenograft assay

1 × 10^7^ cells of A549 stable cell lines (shCtrl/shARHGEF3, shCtrl/shARHGEF3/shARHGEF3+ACLY, Vector/ACLY/K17R /K86R) were mixed in serum-free medium RIPA 1640 (100 μl) and then the cell suspension was inoculated subcutaneously into 4-week-old male nude mice (GemPharmatech CO. Ltd, Nanjing, China) using a 1 ml sterile syringe for in vivo experiments. One month or so, the mice were overanesthetized and killed, and the tumor was taken out for photographing, weighing, and measuring the volume. volume (mm^3^) = π/6 × (large diameter) × (smaller diameter)^2^. The mice were fed in SPF animal Center of Nanchang University.

### Statistical analysis

Each experiment was performed in three independent replicates. Data are shown as means ± SD. *t*-tests were used for statistical comparisons between the two groups. *P* ≤ 0.05 was considered to be statistically significant, *p* < 0.05 (*), *p* < 0.01 (**), *p* < 0.001 (***), and *p* < 0.0001 (****). The statistical data were analyzed by Graphpad Prism software.

## Results

### ARHGEF3 knockdown attenuates the proliferation and tumorigenicity of NSCLC cells

**Fig. 1:**
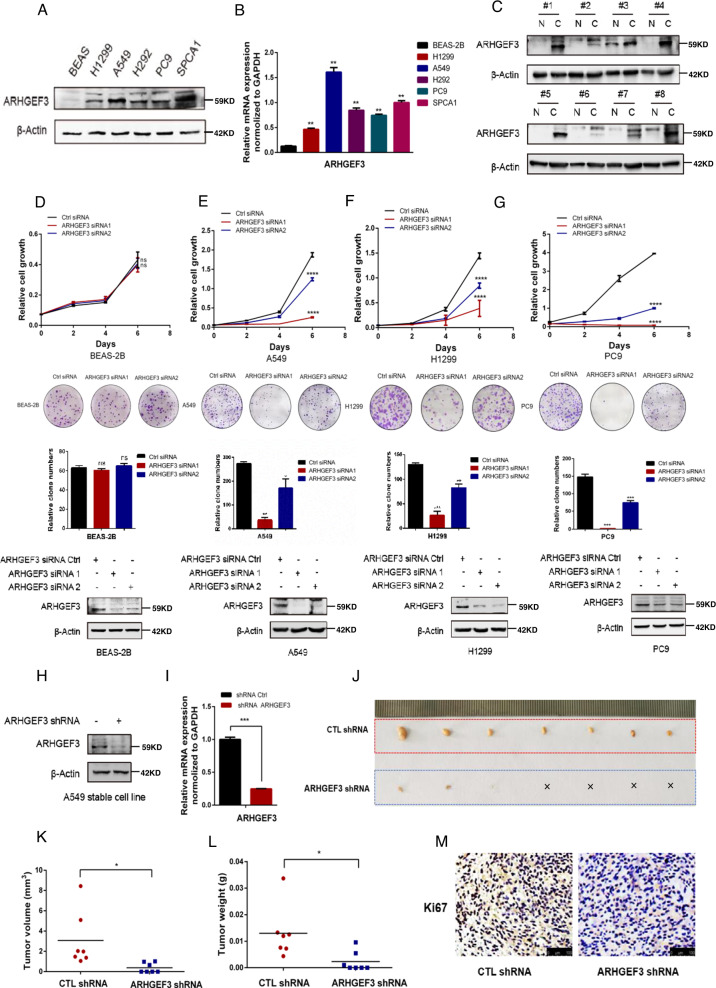
ARHGEF3 knockdown attenuates the proliferation and tumorigenicity of NSCLC cells. **A** Protein levels of ARHGEF3 in H1299, A549, H292, PC9, SPC-A1, and BEAS-2B were determined by western blot with the indicated antibodies. **B** The mRNA level of ARHGEF3 in H1299, A549, H292, PC9, SPC-A1, and BEAS-2B were checked by qPCR. Data represented as the average of three independent experiments (mean ± SD), **P < 0.01. **C** Western blot to detect ARHGEF3 protein levels in paired NSCLC tissues and adjacent tissues (*n* = 8), N normal tissue, C cancer tissue. **D**–**G** The cell growth of human lung epithelial cells BEAS-2B (**D**) and NSCLC cells A549 (**E**), H1299 (**F**), PC9 (**G**) with or without ARHGEF3 knockdown were detected (upper panel), the results represent the average of three independently repeated experiments (mean ± SD), ns no significance, ****P < 0.0001. Knockdown of ARHGEF3 for clonal colony formation assay and quantitative analysis of colony formation assays were performed using ImageJ software (middle panel), the results represent the average of three independently repeated experiments (mean ± SD), ns no significance, *P < 0.05, **P < 0.01, ***P < 0.001. Western blot was used to detect the interference effect of ARHGEF3 siRNA (bottom panel). **H**, **I** The knockdown effect of ARHGEF3 in A549 stable cell line was checked via western blot (**H**) and qPCR (**I**), the results represent the average of three independently repeated experiments (mean ± SD), ***P < 0.001. **J** The xenograft tumor model was constructed by A549 stable cell line with or without ARHGEF3 knockdown. **K**, **L** The volume and weight of xenograft tumors, *p*-value was calculated by paired *t*-test (mean ± SD, *n* = 7), *P < 0.05. **M** IHC of xenograft tumors, Scale bars: 100 μm.

To investigate the functions of ARHGEF3, we first checked the expression of ARHGEF3 in NSCLC cells. Compared with human bronchial epithelial cells BEAS-2B, the protein and mRNA expression of ARHGEF3 were increased in NSCLC cells (Fig. 1A, B). The expression of ARHGEF3 in 8 NSCLC patients was also examined and showed that ARHGEF3 expressed at a higher level in most cancer tissues than in adjacent normal tissues (Fig. 1C). Subsequently, we investigated the function of ARHGEF3 in NSCLC cells. ARHGEF3 was knocked down by specific siRNAs, and cell proliferation assays were performed. The results indicated that knocking down ARHGEF3 did not or only slightly inhibited the proliferation of BEAS-2B cells, but the proliferation rate and colony-forming ability of NSCLC cells were significantly decreased (Fig. 1D–G). The flow cytometry experiments showed that ARHGEF3 knockdown resulted in cell cycle arrest in G0/G1 phase in NSCLC cells, but not in BEAS-2B cells (Fig. S1A–D). Collectively, these results demonstrated that ARHGEF3 knockdown inhibited cell proliferation.

To further confirm the function of ARHGEF3 in NSCLC, we constructed A549 stable cell lines with ARHGEF3 knockdown (Fig. 1H, I). The results showed that ARHGEF3 knockdown had an inhibitory effect on NSCLC cell proliferation (Fig. S1E, F). Moreover, we established a xenograft model to investigate the effect of ARHGEF3 on tumor growth in vivo. The results demonstrated that knockdown of ARHGEF3 led to slower tumor growth (Fig. 1J) and significantly reduced tumor volume (Fig. 1K) and tumor weight (Fig. 1L), and the cells with ARHGEF3 knockdown even could not form obvious xenografts in some models. In clinicopathology, Ki67/MKI67 is commonly referred to as a marker of cancer cell proliferation [14]. The result demonstrated that the expression of Ki67 was decreased in ARHGEF3-knockdown tumors (Fig. 1M), indicating that ARHGEF3 knockdown inhibited cell proliferation and tumor growth in vivo. Taken together, the above results confirmed that ARHGEF3 contributed to the proliferation and tumorigenicity of NSCLC cells.

### ARHGEF3 increases fatty acid synthesis through stabilizing ACLY

**Fig. 2:**
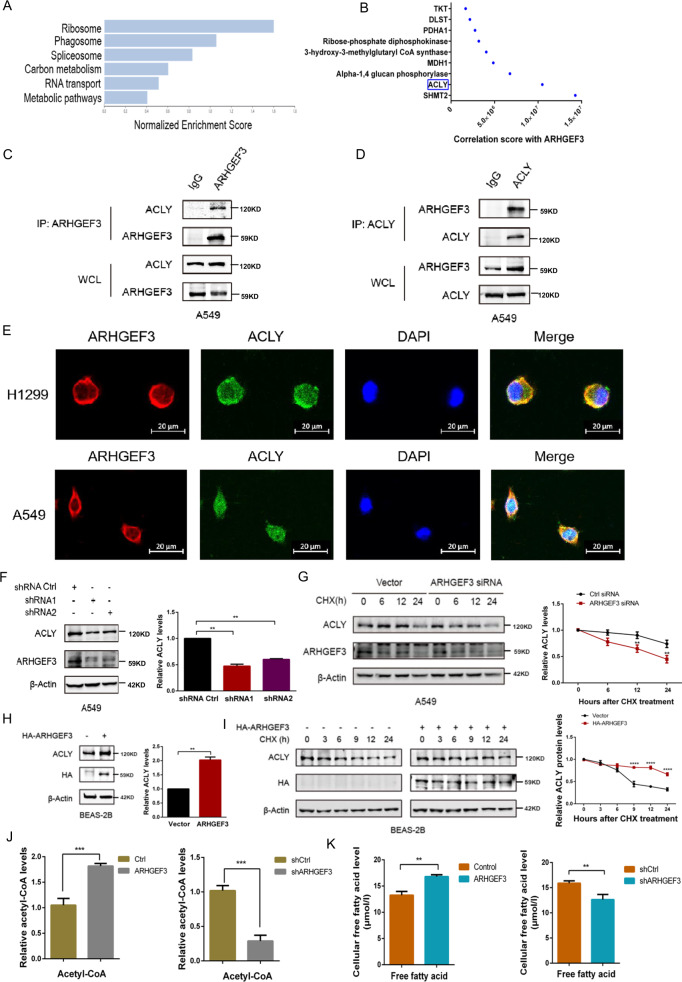
ARHGEF3 increases fatty acid synthesis through stabilizing ACLY. **A** Based on the results of mass spectrometry, the Gene Set Enrichment Analysis (GSEA) of ARHGEF3 interaction protein. **B** The top nine ARHGEF3 interacting proteins from mass spectrometry. **C**, **D** Endogenous interaction between ARHGEF3 and ACLY. **E** The colocalization of ARHGEF3 and ACLY was detected by immunofluorescence assay. **F** shRNA was used to knock down ARHGEF3 and then ACLY protein level was determined by western blot (left). Quantitative analysis of ACLY protein level (right). The results represent the average of three independently repeated experiments (mean ± SD), **P < 0.01. **G** Western blot was used to detect the effect of ARHGEF3 knockdown on the degradation rate of ACLY (left). Quantitative curve analysis of ACLY protein level (right). The results represent the average of three independently repeated experiments (mean ± SD), **P < 0.01. **H** Overexpressing ARHGEF3 in BEAS-2B and then ACLY protein level was determined by western blot (left). Quantitative analysis of ACLY protein level (right). The results represent the average of three independently repeated experiments (mean ± SD), **P < 0.01. **I** Western blot was used to detect the effect of ARHGEF3 overexpression on the degradation rate of ACLY in BEAS-2B cells (left). Quantitative curve analysis of ACLY protein level (right). The results represent the average of three independently repeated experiments (mean ± SD), ****P < 0.0001. **J** ARHGEF3 was overexpressed or knocked down in A549 cells, and the acetyl-CoA content was detected using Acetyl-Coenzyme A assay kit. The results represent the average of three independently repeated experiments (mean ± SD), ***P < 0.001. **K** ARHGEF3 was overexpressed or knocked down in A549 cells, and the fatty acid content was detected using the Human FFA Kit. The results represent the average of three independently repeated experiments (mean ± SD), **P < 0.01.

To explore the mechanism of ARHGEF3 promoting the proliferation of NSCLC cells, we performed mass spectrometry analysis to discover the potential target of ARHGEF3. Interestingly, nine proteins that related to metabolism were identified by gene enrichment pathway analysis. SHMT2 and ACLY were the most potential proteins that interacted with ARHGEF3 (Fig. 2A, B). Immunoprecipitation demonstrated that ARHGEF3 interacted with ACLY (Fig. 2C, D, Fig. S2A, S2B) rather than SHMT2 (Fig. S2C). ATP-citrate lyase (ACLY) has been reported to be abnormally high expressed in many cancers and promotes the proliferation of tumor cells [15–18]. To further validate their interaction, immunofluorescence assays were performed. The results showed that ARHGEF3 co-located with ACLY in the cytoplasm (Fig. 2E), indicating that ARHGEF3 interacted with ACLY.

We next want to explore the relationship between ARHGEF3 and ACLY. First, ARHGEF3 was knocked down by specific siRNAs, then the expression levels of ACLY were detected. The results showed that ARHGEF3 knockdown led to a decrease in ACLY protein levels (Fig. 2F), while the RNA level of ACLY was not affected by ARHGEF3 (Fig. S2D, E), suggesting that ARHGEF3 may affect the protein stability of ACLY. Furthermore, we checked the effect of ARHGEF3 on ACLY protein homeostasis. Cycloheximide (CHX) was used to block the protein synthesis of ACLY, the result revealed that depletion of ARHGEF3 accelerated the degradation of ACLY (Fig. 2G). In addition, the effects of ARHGEF3 on ACLY protein expression were also determined in BEAS-2B cells (where ARHGEF3 is normally expressed at low levels). The result showed that ARHGEF3 overexpression increased the protein level of ACLY (Fig. 2H). We next examined the degradation rates of ACLY in BEAS-2B. The result revealed that overexpression of ARHGEF3 attenuated the degradation of ACLY (Fig. 2I). Subsequently, we detected whether the localization of ACLY changed upon ARHGEF3 knockdown/overexpression, the nucleoplasmic isolation and fluorescence colocalization experiments showed that knockdown or overexpression of ARHGEF3 did not affect ACLY localization (Fig. S2F–K).

Given that ACLY is a cytosolic enzyme that converts citrate into acetyl-CoA [19], which is a precursor for the fatty acid synthesis pathway, we examined the effects of ARHGEF3 on fatty acid metabolism. ARHGEF3 overexpression increased the content of acetyl-CoA, whereas ARHGEF3 knockdown led to a remarkable decrease in acetyl-CoA (Fig. 2J). Consistent with this, the fatty acid synthesis assays indicated that the overexpression of ARHGEF3 increased the content of fatty acid, conversely, ARHGEF3 knockdown attenuated fatty acid synthesis (Fig. 2K). In conclusion, these results demonstrated that ARHGEF3 stabilized ACLY protein to promote fatty acid synthesis in NSCLC cells.

### ARHGEF3 enhances the interaction between ACLY and SIRT2

**Fig. 3:**
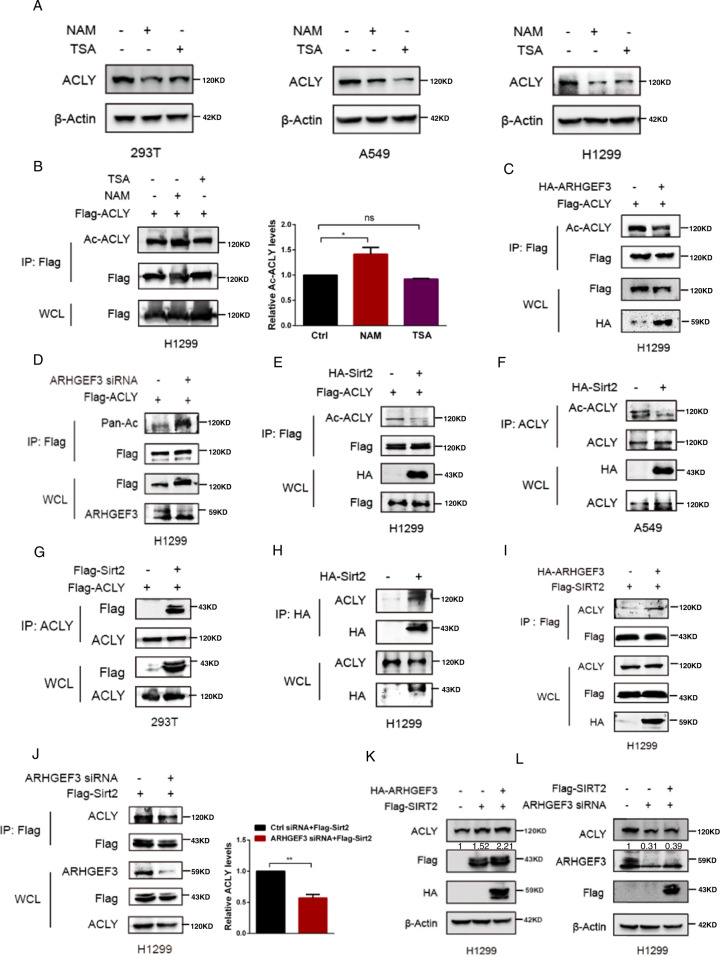
ARHGEF3 enhances the interaction between ACLY and SIRT2. **A** Western blot was used to detect ACLY protein levels after NAM and TSA treatment. **B** Western blot was used to detect ACLY acetylation levels after NAM and TSA treatment (left). Quantitative analysis of ACLY protein level (right). The results represent the average of three independently repeated experiments (mean ± SD), ns no significance, *P < 0.05. **C** Western blot was used to detect the ACLY acetylation after ARHGEF3 overexpression. **D** Western blot was used to detect the ACLY acetylation after ARHGEF3 knockdown. **E**, **F** Western blot was used to detect the effect of SIRT2 on ACLY acetylation. **G**, **H** HEK293T and H1299 cells were transfected with indicated plasmids and immunoprecipitation was performed to detect SITR2-ACLY interaction. **I** Western blot was used to detect the influence of overexpression of ARHGEF3 on SIRT2-ACLY interaction. **J** Western blot was used to detect the influence of ARHGEF3 knockdown on SIRT2-ACLY interaction (left). Quantitative analysis of ACLY protein level (right). The results represent the average of three independently repeated experiments (mean ± SD), **P < 0.01. **K** Western blot was used to detect the influence of overexpression of ARHGEF3 and SIRT2 on ACLY protein level. **L** Western blot was used to detect the influence of ARHGEF3 knockdown and SIRT2 on ACLY protein level.

Previous studies demonstrated that ACLY protein homeostasis was regulated by acetylation [20, 21]. We wondered whether ARHGEF3 affected ACLY protein stability via regulating acetylation. The protein expression of ACLY was decreased when treating cells with NAM or TSA, two deacetylase inhibitors of the SIRT and HDAC class I/II families (Fig. 3A). These results suggested that SIRT or HDAC class I/II family proteins might be related to ACLY protein homeostasis. To distinguish the roles of these two types of deacetylases, we treated cells with NAM and TSA followed by detecting the acetylation of ACLY. We found that NAM treatment increased ACLY acetylation but not TSA (Fig. 3B), indicating that the acetylation and stability of ACLY protein might be regulated by SIRTs. To distinguish the effects of TSA on the expression of ACLY, we detected the mRNA level of ACLY in H1299 and A549 cells treated with TSA. Figure S2L showed that TSA treatment had no significant effect on the mRNA level of ACLY, indicating that TSA did not affect ACLY expression through transcription. Previous studies have shown that HDAC class I/II also regulates other protein acylation except for acetylation [22–24]. Thus, we speculated that the effect of TSA on ACLY expression might be caused by the regulation of other types of protein acylation rather than acetylation, and we will conduct this research on another independent subject. Subsequently, we attempted to test the relationship between ARHGEF3 and SIRTs. Immunoprecipitation assays showed that ARHGEF3 interacted with SIRTs distributed in the cytoplasm (SIRT1, SIRT2, SIRT5, SIRT6, SIRT7) and was irrelevant to HDACs (Fig. S3A–K). Ectopic ARHGEF3 expression decreased ACLY acetylation levels in H1299 cells (Fig. 3C), while siRNA-mediated knockdown of ARHGEF3 increased its acetylation (Fig. 3D). SIRT2 is reported to be responsible for ACLY acetylation. As shown in Fig. 3E and Fig. S3L–Q, ectopic SIRT2 expression significantly decreased ACLY acetylation levels, but not other SIRTs. Besides, SIRT2 overexpression also reduced endogenous ACLY acetylation levels (Fig. 3F), and SIRT2 interacted with ACLY (Fig. 3G, H). These results revealed that ACLY acetylation level was mainly mediated by SIRT2.

Interestingly, overexpressing ARHGEF3 facilitated the interaction between SIRT2 and ACLY (Fig. 3I, Fig. S3R), by contrast, ARHGEF3 knockdown attenuated this effect (Fig. 3J, Fig. S3S). Then, we explored whether ARHGEF3 regulates ACLY protein homeostasis through SIRT2. Ectopic SIRT2 expression increased ACLY protein level, and the upregulation of ACLY protein level was more obvious when ARHGEF3 was overexpressed (Fig. 3K). To further validate this, siRNAs were used to knock down ARHGEF3 expression, the results showed that knocking down ARHGEF3 resulted in a decrease in ACLY protein level, and overexpressing SIRT2 under this condition could not increase ACLY expression (Fig. 3L). Together with the data above, these results indicated that ARHGEF3 affected ACLY protein homeostasis by facilitating SIRT2-ACLY interaction.

### The K17 and K86 were essential acetylation sites of ACLY for ARHGEF3 to recruit SIRT2 for deacetylating and stabilizing ACLY

**Fig. 4:**
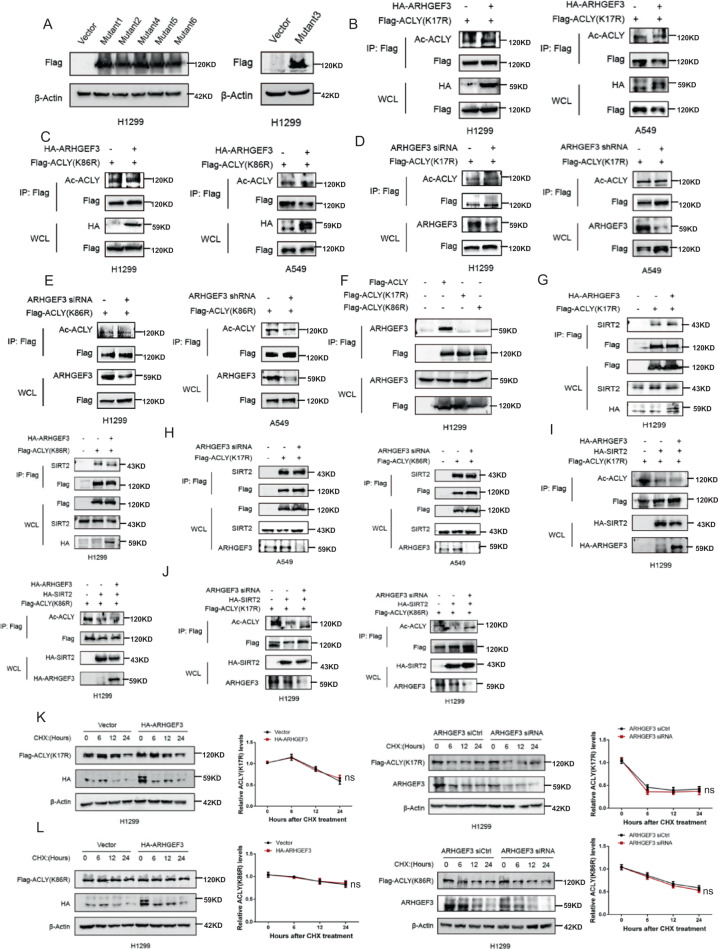
The K17 and K86 were essential acetylation sites of ACLY for ARHGEF3 to recruit SIRT2 for deacetylating and stabilizing ACLY. **A** Six ACLY acetylation-deficient mutants. **B**, **C** H1299 and A549 cells were co-transfected with pcDNA3.1-HA-ARHGEF3, and ACLY mutants, the acetylation of ACLY-K17R and K86R was checked by immunoprecipitation and western blot. **D**, **E** H1299 and A549 cells were co-transfected with ARHGEF3 siRNA or shRNA and ACLY mutants, the acetylation of ACLY-K17R and 86 R was checked by immunoprecipitation and western blot. **F** The interaction between ARHGEF3 and ACLY, ACLY-K17R, 86 R. **G** The effect of ARHGEF3 overexpression on SIRT2-ACLY(K17R)/(K86R) interaction was detected by western blot. **H** The effect of ARHGEF3 knockdown on SIRT2-ACLY (K17R)/(K86R) interaction was detected by western blot. **I** H1299 cells were transfected with Flag-tagged SIRT2 and Flag-tagged ACLY-K17R or 86 R and HA-tagged ARHGEF3. The acetylation of ACLY-K17R and 86R were determined by western blot. **J** Flag-tagged SIRT2, Flag-tagged ACLY-K17R or 86R and ARHGEF3 siRNA were co-transfected in H1299 cells. Western blot was used to check the acetylation of ACLY-K17R and 86R. **K**, **L** Cycloheximide (CHX) (25 μg/ml) was incubated for the indicated period with H1299 cells transfected with ACLY mutants and pcDNA3.1-HA-ARHGEF3 or ARHGEF3 siRNA. Western blot was used to detect the effects of ARHGEF3 on the degradation rates of ACLY-K17R and 86 R (left). Degradation curve ACLY-K17R and 86 R (right). The results represent the average of three independently repeated experiments (mean ± SD), ns no significance.

To further clarify the mechanism of ARHGEF3 in regulating ACLY acetylation, we first searched for the potential acetylation sites of ACLY. Proteomic analysis based on mass spectrometry revealed ten putative acetylation sites of ACLY (K17, K86, K540, K546, K554, K948, K962, K968, K978, and K1077) [20]. To assess whether these acetylation sites are regulated by ARHGEF3, we constructed six acetylation-deficient mutants from lysine to arginine (K17R, K86R, K540R/546 R/554 R, K948R/962 R, K968R/978 R, and K1077R), named mutants 1–6 (Fig. 4A). Subsequently, we detected the effect of ARHGEF3 on the acetylation of ACLY mutants. The results showed that the acetylation levels of ACLY-K17R (Fig. 4B) and ACLY-K86R (Fig. 4C) were almost unchanged when ARHGEF3 was overexpressed. To further valid this, we used specific siRNAs to knock down ARHGEF3, the results demonstrated that the absence of ARHGEF3 had no effect on ACLY-K17R acetylation (Fig. 4D) and ACLY-K86R (Fig. 4E). Besides, overexpression of ARHGEF3 could affect the acetylation levels of ACLY-K540R/546 R/554 R and ACLY-K948R/962 R, but had little effect on ACLY- K968R/978 R and ACLY-K1077R (Fig. S4A–D), while knockdown of ARHGEF3 could significantly enhance the acetylation levels of ACLY-K540R/546 R/554 R, ACLY-K948R/962 R, ACLY-K968R/978 R and ACLY-K1077R (Fig. S4E–H). In addition, immunoprecipitation results revealed that ARHGEF3 interacted with wild-type ACLY (ACLY-WT), but not with ACLY-K17R and 86 R (Fig. 4F), indicating that acetylation on K17 or K86 of ACLY was essential for its interaction with ARHGEF3. We next detected the effect of ARHGEF3 on the interaction between SIRT2 and ACLY (K17R/K86R). We found that overexpression or knockdown of ARHGEF3 did not affect the interaction between ACLY (K17R/K86R) and SIRT2 (Fig. 4G, H). Furthermore, the overexpression of SIRT2 decreased the acetylation of ACLY-K17R and ACLY-K86R, while simultaneous ectopic ARHGEF3 expression failed to significantly strengthen the deacetylation of these two mutants (Fig. 4I). To further prove this, siRNAs were used to knock down ARHGEF3 expression, the results showed that the decreased acetylation of ACLY-K17R and 86R induced by SIRT2 overexpression did not recover after ARHGEF3 knockdown (Fig. 4J). ACLY-3K (K540/546/554) was reported to be deacetylation sites mediated by SIRT2, and we found that SIRT2 could still deacetylate ACLY-3K mutant (Fig. S4I, J). This indicated that SIRT2 had many deacetylation sites on ACLY. Considering that ARHGEF3 affects the stability of ACLY, we checked the half-life of ACLY-K17R and 86 R. The results confirmed that the half-life of ACLY-K17R and 86 R were not affected by ARHGEF3 overexpression or knockdown (Fig. 4K, L). Together with the data above, we concluded that ARHGEF3 tends to interact with acetylated ACLY on K17 and K86, recruiting SIRT2 to deacetylate and stabilize ACLY.

### ARHGEF3 affects NEDD4-mediated ubiquitination of ACLY

**Fig. 5:**
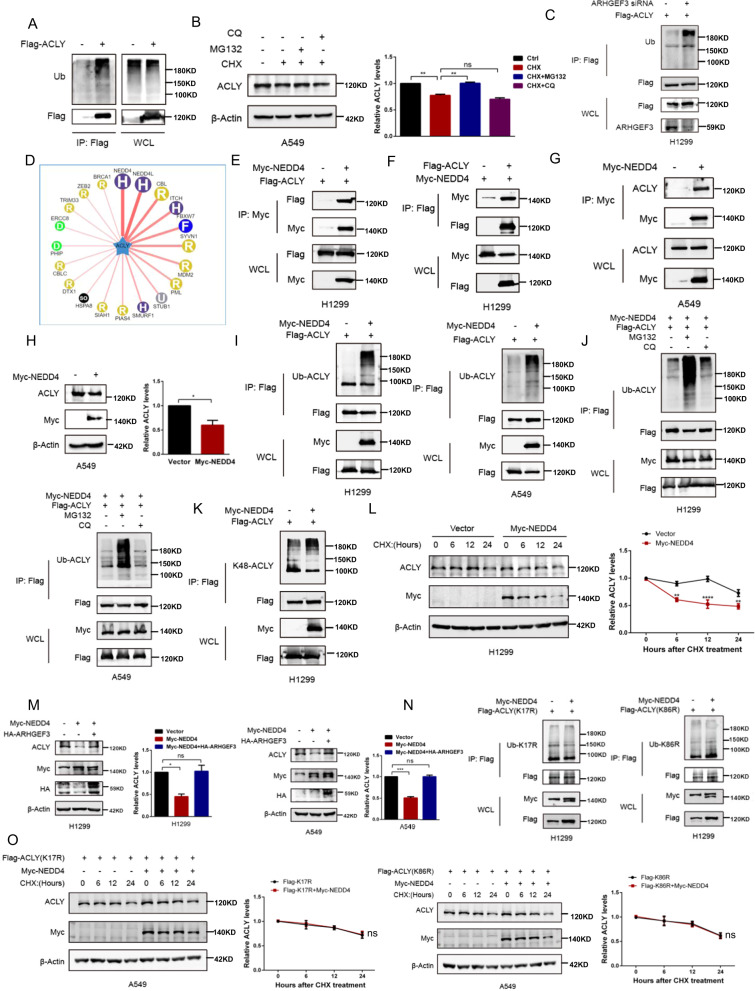
ARHGEF3 promotes the stability of ACLY through inhibiting NEDD4-mediated ubiquitination. **A** The ubiquitination level of ACLY. Flag-tagged ACLY was transfected in H1299 cells. Ubiquitylation of immunoprecipitated ACLY was determined. **B** Western blot analysis of ACLY protein level after MG132 (10 μM) and CQ (20 μM) treated for 12 h (left panel). Quantitative statistical analysis between groups (right panel). The results represent the average of three independently repeated experiments (mean ± SD), ns no significance, **P < 0.01. **C** Western blot was used to detect the ubiquitination level of ACLY after ARHGEF3 knockdown. **D** Predicted E3 kinase of ACLY. **E**–**G** The interaction between NEDD4 and ACLY. **H** Western blot was used to detect the effect of NEDD4 on ACLY protein level (left). Quantitative analysis of ACLY protein level (right). The results represent the average of three independently repeated experiments (mean ± SD), *P < 0.05. **I** Western blot was used to detect the effect of NEDD4 on ACLY ubiquitin level. **J** Western blot was used to detect the effect of NEDD4 on ACLY ubiquitin level after MG132 and CQ treatment. **K** Western blot was used to detect the effect of NEDD4 on the K48-ubiquitination level of ACLY. **L** Western blot was used to detect the effect of NEDD4 on ACLY degradation rate (left). The degradation curve of ACLY (right). The results represent the average of three independently repeated experiments (mean ± SD), **P < 0.01, ****P < 0.0001. **M** Western blot was used to detect the effect of NEDD4 on ACLY protein level after overexpression of ARHGEF3 (left). Quantitative analysis of ACLY protein level (right). The results represent the average of three independently repeated experiments (mean ± SD), ns no significance, *P < 0.05, ***P < 0.001. **N** Western blot was used to detect the effect of NEDD4 on ACLY-K17R and 86 R ubiquitination levels. **O** Western blot was used to detect the effect of NEDD4 on ACLY-K17R and 86 R degradation rate (left). The degradation curve of ACLY-K17R and 86 R (right). The results represent the average of three independently repeated experiments (mean ± SD), ns no significance.

Our previous results in Fig. 2 showed that ARHGEF3 affects ACLY protein stability, and we next detected how ACLY was degraded. We found that ACLY was actively ubiquitinated (Fig. 5A), and the proteasome inhibitor MG132 blocked ACLY degradation but not chloroquine (CQ) (Fig. 5B), suggesting that ACLY is degraded through the proteasome pathway. Subsequently, we examined the effect of ARHGEF3 on ACLY ubiquitination, and the results showed that the ubiquitination of ACLY was significantly increased after ARHGEF3 knockdown (Fig. 5C). To clarify how ARHGEF3 regulates the stability of ACLY, we used bioinformatics to analyze the amino acid sequence of ARHGEF3, but did not find any conserved E3-like domain (data not shown). Therefore, we supposed that ARHGEF3 might affect ACLY ubiquitination by regulating a certain E3 ligase. The predicted E3 ligases of ACLY were found on the UbiBrowser website (http://ubibrowser.ncpsb.org.cn/ubibrowser/) (Fig. 5D). Neuronally expressed developmentally downregulated 4 (also called NEDD4) was considered to be the preferred E3 ligase for ACLY because of the highest score. NEDD4 is a HECT E3 ubiquitin ligase [25], but its role in mediating ACLY ubiquitination has not been reported. The interaction between ACLY and NEDD4 was demonstrated by Co-IP assays (Fig. 5E–G), and ectopic NEDD4 expression decreased ACLY protein level in A549 cells (Fig. 5H). Subsequently, we asked whether NEDD4 was necessary for the ubiquitination of ACLY. Indeed, NEDD4 regulated ACLY ubiquitination (Fig. 5I). In addition, NEDD4 significantly increased the ubiquitination of ACLY after MG132 treatment, but not CQ (Fig. 5J). These results revealed that ACLY was ubiquitinated by NEDD4 and degraded via the proteasome pathway. Previous studies reported that the K48-linked ubiquitination was usually a protein degradation signal, and its substrate proteins entered the proteasome for degradation [26, 27]. We further detected the K48-linked ubiquitination of ACLY. As shown in Fig. 5K, NEDD4 overexpression enhanced K48-linked ubiquitination of ACLY. To assess whether NEDD4 affects ACLY stability, blocking protein synthesis with CHX revealed that ectopic NEDD4 expression accelerated the degradation of ACLY (Fig. 5L). These results demonstrated that NEDD4 promoted ACLY degradation by mediating K48-linked ubiquitination. Furthermore, we detected whether ARHGEF3 affected the NEDD4-mediated ubiquitination of ACLY. As a result, ectopic ARHGEF3 expression increased ACLY protein levels by attenuating NEDD4-mediated ACLY degradation in H1299 and A549 cells (Fig. 5M).

To determine whether there is a definite connection between deacetylation and ubiquitination, we checked the ubiquitination levels of ACLY-K17R and 86 R. The results showed that NEDD4 has no interaction with 17 R and 86 R (Fig. S5A, B). And the ubiquitination of these two mutants was almost unchanged when overexpressing NEDD4 (Fig. 5N). We then used cycloheximide (CHX) to block protein synthesis and tested the stability of ACLY-K17R and 86 R under physiological conditions in A549 and H1299 cells. The stability of ACLY-17R and 86 R were not significantly altered after NEDD4 overexpression (Fig. 5O, Fig. S5C, D). Taken together, these results demonstrated that NEDD4 promoted the degradation of ACLY via mediating K48-linked ubiquitination, and ACLY deacetylation inhibited NEDD4-mediated degradation.

### ARHGEF3 regulates ACLY stability independently of its GEF activity

**Fig. 6:**
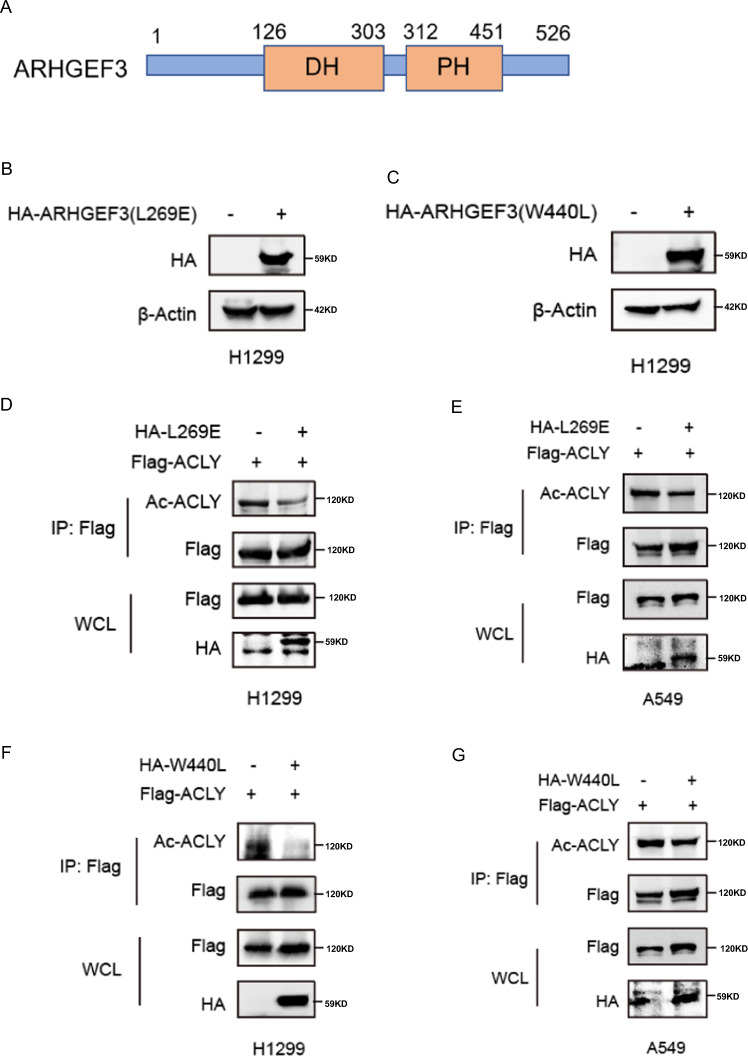
ARHGEF3 regulates ACLY stability independently of its GEF activity. **A** Structural diagram of ARHGEF3. **B**, **C** Western blot was used to detect the expression effect of ARHGEF3-L269E and W440L. **D**, **E** Western blot was used to detect the effect of ARHGEF3-L269E on ACLY acetylation. **F**, **G** Western blot was used to detect the effect of ARHGEF3-W440L on ACLY acetylation.

Since ARHGEF3 functions as a Rho GEF, we investigated whether GEF activity of ARHGEF3 participates in ACLY regulation. It has been reported that ARHGEF3-L269E and W440L were each sufficient to inactivate GEF activity (Fig. 6A) [28]. Therefore, inactivated mutants ARHGEF3-L269E and W440L were constructed to detect the effect on ACLY acetylation (Fig. 6B, C). Figure 6D and E showed that ectopic ARHGEF3-L269E expression decreased the acetylation of ACLY in H1299 and A549 cells in the same way as WT-ARHGEF3 and similar results were also observed in W440L mutant (Fig. 6F, G). These results confirmed that ARHGEF3 regulated the acetylation of ACLY independently of its GEF3 activity.

### ARHGEF3 promotes NSCLC cell proliferation by regulating ACLY

**Fig. 7:**
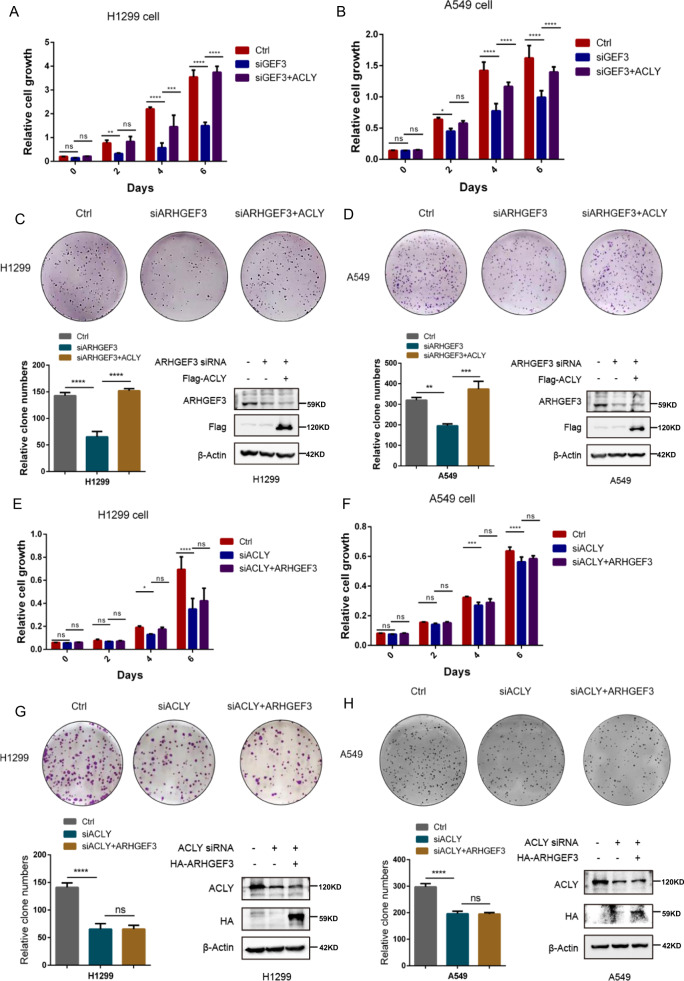
ARHGEF3 promotes NSCLC cell proliferation by regulating ACLY. **A**, **B** The effects of ACLY on the growth of H1299 and A549 cells after ARHGEF3 knockdown was detected by cell count assays. The results represent the average of three independently repeated experiments (mean ± SD), ns no significance, *P < 0.05, **P < 0.01, ***P < 0.001, ****P < 0.0001. **C**, **D** The effects of ACLY on the proliferation of H1299 and A549 cells after ARHGEF3 knockdown were detected by clonal colony formation assays (upper panel). Statistical analysis between groups (bottom panel), the results represent the average of three independently repeated experiments (mean ± SD), **P < 0.01, ***P < 0.001, ****P < 0.0001. **E**, **F** The effects of ARHGEF3 on the growth of H1299 and A549 cells after ACLY knockdown was detected by cell count assays. The results represent the average of three independently repeated experiments (mean ± SD), ns no significance, *P < 0.05, ***P < 0.001, ****P < 0.0001. **G**, **H** The effects of ARHGEF3 on the proliferation of H1299 and A549 cells after ACLY knockdown were detected by clonal colony formation assays (upper panel). Statistical analysis between groups (bottom panel), the results represent the average of three independently repeated experiments (mean ± SD), ns no significance, ****P < 0.0001.

To further examine whether ARHGEF3 promoted tumor growth through ACLY. siRNA was used to specifically interfere with ARHGEF3 expression in H1299 and A549 cells, and the cell growth assays revealed that ectopic ACLY expression recovered cancer cell proliferation (Fig. 7A, B). In line with this, colony formation experiments demonstrated that overexpression of ACLY could rescue the colony-forming ability of NSCLC cells blocked by ARHGEF3 depletion (Fig. 7C, D). To further valid this, we knocked down ACLY and found that the proliferation of H1299 and A549 cells was attenuated, and ectopic ARHGEF3 expression did not increase cell growth (Fig. 7E, F). The colony formation experiments also confirmed that overexpression of ARHGEF3 did not resist the inhibition of cell proliferation caused by ACLY knockdown (Fig. 7G, H). In conclusion, these results indicated that ARHGEF3 promoted NSCLC cell proliferation by regulating ACLY.

### ARHGEF3 promotes tumorigenesis by regulating the stability of ACLY

**Fig. 8:**
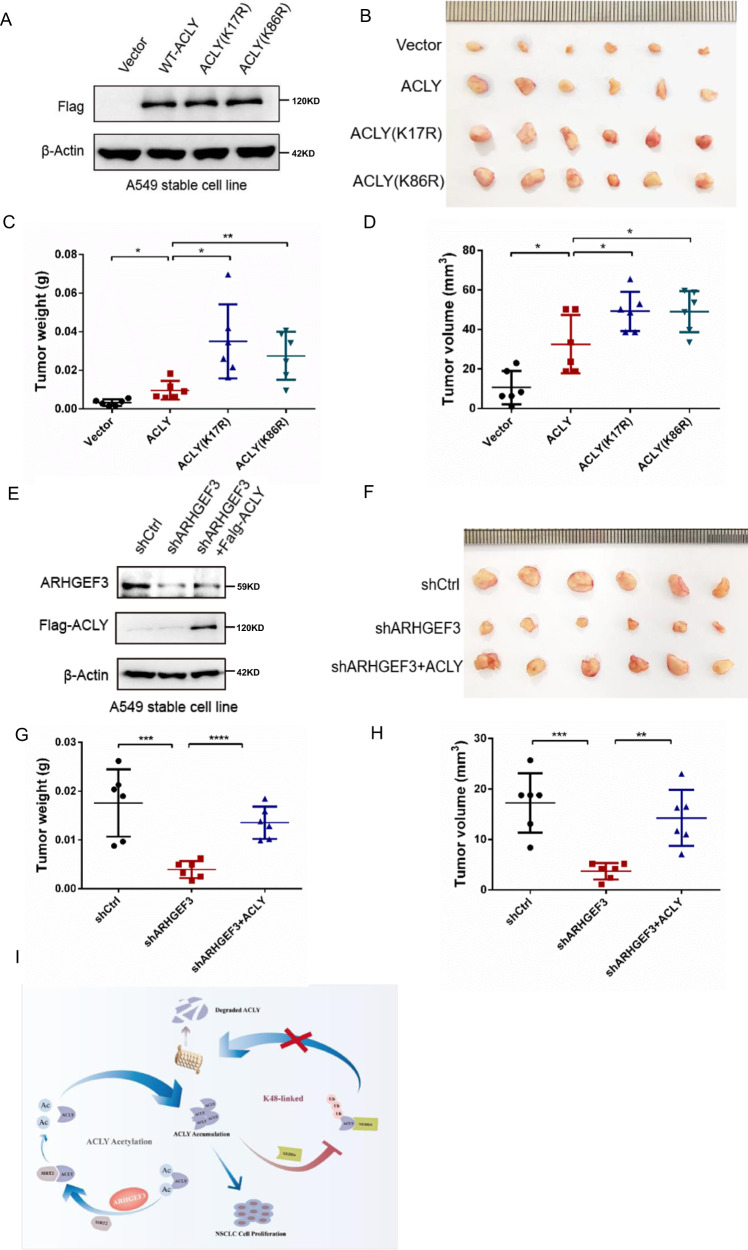
ARHGEF3 promotes tumorigenesis by regulating the stability of ACLY. **A** Detection of expression effect of acetylation-deficient mutants. **B** The xenograft tumor model was constructed by A549 stable cell line with the overexpression of WT-ACLY, ACLY(K17R), and ACLY(K86R). **C**, **D** The weight and volume of xenograft tumors, *p*-value was calculated by paired *t*-test (mean ± SD, *n* = 6). *P < 0.05, **P < 0.01. **E** Detection of expression effect of ARHGEF3 knockdown and ACLY overexpression. **F** The xenograft tumor model was constructed by A549 stable cell line with ARHGEF3 knockdown and ARHGEF3 knockdown combined with ACLY overexpression. **G**, **H** The weight and volume of xenograft tumors, *p*-value was calculated by paired *t*-test (mean ± SD, *n* = 6). **P < 0.01, ***P < 0.001, ****P < 0.0001. **I** The working model of ARHGEF3 in regulating ACLY.

The above results have confirmed that ARHGEF3 promoted cancer cell proliferation and tumor growth. To further clarify that ARHGEF3 promoted tumorigenesis by regulating ACLY, we constructed A549 stable cell lines overexpressing WT-ACLY, ACLY (K17R), and ACLY (K86R), respectively (Fig. 8A). In vivo experiments demonstrated overexpression of ACLY promoted tumor growth and significantly increased tumor weight and volume. As we expected, the tumorigenicity of stable cell lines expressing acetylation-deficient mutants of ACLY was higher than ACLY-WT (Fig. 8B–D). Furthermore, to detect whether overexpression of ACLY could rescue the loss of tumor formation in ARHGEF3-deficient cells, A549 stable cell lines with ARHGEF3 knockdown combined with ACLY overexpression were constructed (Fig. 8E). The results revealed that ARHGEF3 knockdown attenuated tumor growth and obviously reduced tumor weight and volume, but ACLY overexpression could alleviate the tumor-inhibiting effects of ARHGEF3 knockdown (Fig. 8F–H). Taken together, these results indicated that ARHGEF3 promotes tumorigenesis by regulating the stability of ACLY.

## Discussion

Rho GTPases are crucial signal transduction factors that regulate various cellular processes such as cell proliferation, survival, migration, and death. Rho GTPases are essential for maintaining normal cell biological functions, but their dysregulation can also facilitate the progression of cancer [29–31]. Rho GEFs are the positive regulators of GTPase and have been demonstrated to function in tumor initiation and migration. Previous studies have demonstrated that ARHGEF16 enhanced the migration and proliferation of human glioma cells [32]. ARHGEF5 promoted tumor malignancy via epithelial-mesenchymal transition [33]. ARHGEF9 was reported to be DNA methylation biomarker for hepatocellular carcinoma [34]. ARHGEF4 predicts poor prognosis and promotes cell invasion by influencing ERK1/2 and GSK-3/ signaling in pancreatic cancer [35]. ARHGEF39 promotes gastric cancer cell proliferation and migration via Akt signaling pathway [36]. ARHGEF28 promotes colon carcinoma cell motility and tumor progression via interaction with focal adhesion kinase (FAK) [37]. ARHGEF12 is a potential oncogene in acute leukemia [38]. However, the role of ARHGEF3 in NSCLC has not been reported, and the function of ARHGEF3 in cancer remains to be further elucidated.

ACLY is involved in lipid biogenesis and plays a vital role in tumor progression [39]. A previous study has reported that ACLY knockdown impairs cell proliferation by affecting the cell cycle in NSCLC [10]. SIRT2 is reported to be the main deacetylase of ACLY and lysines 540,546 and 554 are the acetylation sites of ACLY [20]. Herein, we found that lysines 17 and 86 are the new acetylation sites of ACLY that were important for its protein homeostasis. ARHGEF3 interacted with acetylated ACLY on K17 and K86, and stabilized ACLY by increasing SIRT2-mediated deacetylation of ACLY. Miaomiao Tian et al. reported that CUL3-KLHL25 mediated ACLY ubiquitination and degradation [40]. UBR4 is another E3 ligase of ACLY [20]. To further explore the regulatory mechanism of ACLY protein homeostasis, we searched for potential E3 ligase based on UbiBrowser website, and NEDD4 was identified. Accumulating evidence indicated that the NEDD4 played an essential role in a variety of cellular processes through the ubiquitination-mediated degradation of multiple substrates [41]. We confirmed that NEDD4 promoted the degradation of ACLY by mediating ubiquitination, which relied on the deacetylation of ACLY, and ARHGEF3 expression blocked NEDD4-mediated degradation of ACLY.

Given ARHGEF3 is a member of Rho GEFs, its known function is to mediate the activation of Rho protein. Therefore, we also investigated whether the regulation of ARHGEF3 on ACLY depended on GEF activity. Based on the previous reports, we constructed GEF inactivated mutants (L269E and W440L), and subsequent experiments proved that it is a novel function of ARHGEF3 to regulate ACLY, which is independent of GEF activity.

In conclusion, our studies unravel a novel function of ARHGEF3 independent of GEF activity in non-small cell lung cancer (Fig. 8I). Highly expressed ARHGEF3 promotes NSCLC cell proliferation and tumorigenicity. The mechanism is that ARHGEF3 regulates the protein homeostasis of ACLY through recruiting SIRT2 to deacetylate ACLY, thus inhibiting NEDD4-mediated ubiquitination. In conclusion, ARHGEF3 is a new potential therapeutic target for non-small cell lung cancer.

## Supplementary information

Supplemental Figure legends

Figure S1

Figure S2

Figure S3

Figure S4

Figure S5

Reproducibility checklist

## Data Availability

All datasets used and analyzed in the current study are available on reasonable request from the corresponding author.
