# Supplementary material for: ARHGEF3 regulates the stability of ACLY to promote the proliferation of lung cancer
Source: Cell Death Dis. 2022 Oct 14;13(10):870. doi: 10.1038/s41419-022-05297-4 (PMC9568610; doi:10.1038/s41419-022-05297-4)
Supplement: Supplementary file 1 — Supplemental Figure legends [file 41419_2022_5297_MOESM1_ESM.docx]

**Supplemental Figure legends**

**Figure S1. ARHGEF3 knockdown inhibits cell proliferation by arresting the cell cycle at G0/G1 phase**

(A-D) Human lung epithelial cells BEAS-2B (A) and NSCLC cell lines A549 (B), H1299 (C) and PC9 (D) were transfected with either Ctrl siRNA or ARHGEF3 siRNAs. After 48 h, cell cycle analysis was done by flow cytometry (left). Quantitative analysis of cell counting (right). The data represent the averages of three independent experiments (mean ± SD), ns, no significance, **P < 0.05, ***P < 0.001, ****P < 0.0001. (E) Growth curves of A549 stable cell line with or without ARHGEF3 knockdown. The data represent the averages of three independent experiments (mean ± SD), ****P < 0.0001. (F) The proliferative ability of A549 stable cell line with or without ARHGEF3 knockdown were assessed by colony formation assays.

**Figure S2.** **The mRNA level of ACLY is not regulated by ARHGEF3**

(A, B) H1299 and A549 cells were transfected with HA-ARHGEF3 plasmids, the interactions between ARHGEF3 and ACLY were determined by immunoprecipitation. (C) HEK293T cells were transfected with HA-ARHGEF3 or HA-SHMT2 plasmids, the interactions between ARHGEF3 and SHMT2 were determined by immunoprecipitation. (D, E) A549 and H1299 cells were transfected with ARHGEF3 plasmids or ARHGEF3 siRNAs, the mRNA levels of ACLY were detected by qPCR. (F, G) ACLY localization was detected by overexpression of ARHGEF3 in H1299 and A549 cells (left). Quantitative analysis of ACLY protein levels (right). (H, I) ACLY localization was detected by ARHGEF3 knockdown in H1299 and A549 cells (left). Quantitative analysis of ACLY protein levels (right). (J, K) The localization of ACLY was detected by immunofluorescence assay in H1299 and A549 cells overexpressing or knocking down ARHGEF3. (L) H1299 and A549 cells were treated with TSA to detect ACLY mRNA levels. The data represent the averages of three independent experiments (mean ± SD), ns, no significance.

**Figure S3. Interactions of ARHGEF3 with SIRTs and HDACs**

(A-G) The interactions between ARHGEF3 and SIRTs (SIRT1, SIRT2, SIRT5, SIRT6, SIRT7) were determined by immunoprecipitation. (H-K) H1299 cells were co-transfected with HA-ARHGEF3 and HA-HDAC4 (H), or HA-HDAC6 (I), HA-HDAC7 (J), HA-HDAC10 (K). The interactions between ARHGEF3 and HDACs were determined by immunoprecipitation. (L-Q) Western blot was used to detect the effect of SIRT1, SIRT3, SIRT4, SIRT5, SIRT6 and SIRT7 on ACLY acetylation. (R) H1299 cells were co-transfected with HA-ARHGEF3 and Flag-SIRT2 or Vector, 48h later. Proteins were immunoprecipitated with Flag antibody. The interaction between SIRT2 and ACLY were determined by western blot. (S) H1299 cells were co-transfected with ARHGEF3 shRNA and Flag-SIRT2 or Vector, 48h later. Proteins were immunoprecipitated with Flag antibody. The acetylation of ACLY were determined by western blot.

**Figure S4. ARHGEF3 expression affect the acetylation of other ACLY mutations except K17 and K86**

(A-D) H1299 cells were transfected with HA-ARHGEF3 and Flag-ACLY-K540R/546R/554R (A) or K948R/K962R (B), K968R/K978R (C), K1077R (D), the acetylation of ACLY mutants were detected by immunoprecipitation and western blot. (E-H) H1299 cells were transfected with ARHGEF3 siRNA and Flag-ACLY-K540R/546R/554R (E) or K948R/K962R (F), K968R/K978R (G), K1077R (H), the acetylation of ACLY mutants were detected by immunoprecipitation and western blot. (I) The interaction between SIRT2 and ACLY-3R (K540R/546R/554R). (J) The acetylation of ACLY-3R was detected by overexpression of SIRT2.

**Figure S5.** **Acetylation on K17 or K86 is essential for NEDD4-mediated degradation of ACLY**

(A) 293T cells were co-transfected with Flag-ACLY-K17R and Myc-NEDD4 or Vector, 48h later, the interaction between NEDD4 and K17R was detected by immunoprecipitation. (B) 293T cells were co-transfected with Flag-ACLY-K86R and Myc-NEDD4 or Vector, 48h later, the interaction between NEDD4 and K86R was detected by immunoprecipitation. (C) H1299 cells were co-transfected with Flag-ACLY-K17R and Myc-NEDD4 or Vector, 36h later, cells were treated with 25 μg/ml CHX at indicated time. The ACLY-K17R expression was detected by western blot (left). ACLY-K17R expression relative to Actin was quantified (right). Data represents the average of three independent experiments (mean± SD), ns, no significance. (D) H1299 cells were co-transfected with Flag-ACLY-K86R and Myc-NEDD4 or Vector, 36h later, cells were treated with 25 μg/ml CHX at indicated time. The ACLY-K17R expression was detected by western blot (left). ACLY-K86R expression relative to Actin was quantified. Data represents the average of three independent experiments (mean± SD) (right), ns, no significance.
