# Supplementary figures and images for: ARHGEF3 regulates the stability of ACLY to promote the proliferation of lung cancer
Source: Cell Death Dis. 2022 Oct 14;13(10):870. doi: 10.1038/s41419-022-05297-4 (PMC9568610; doi:10.1038/s41419-022-05297-4)

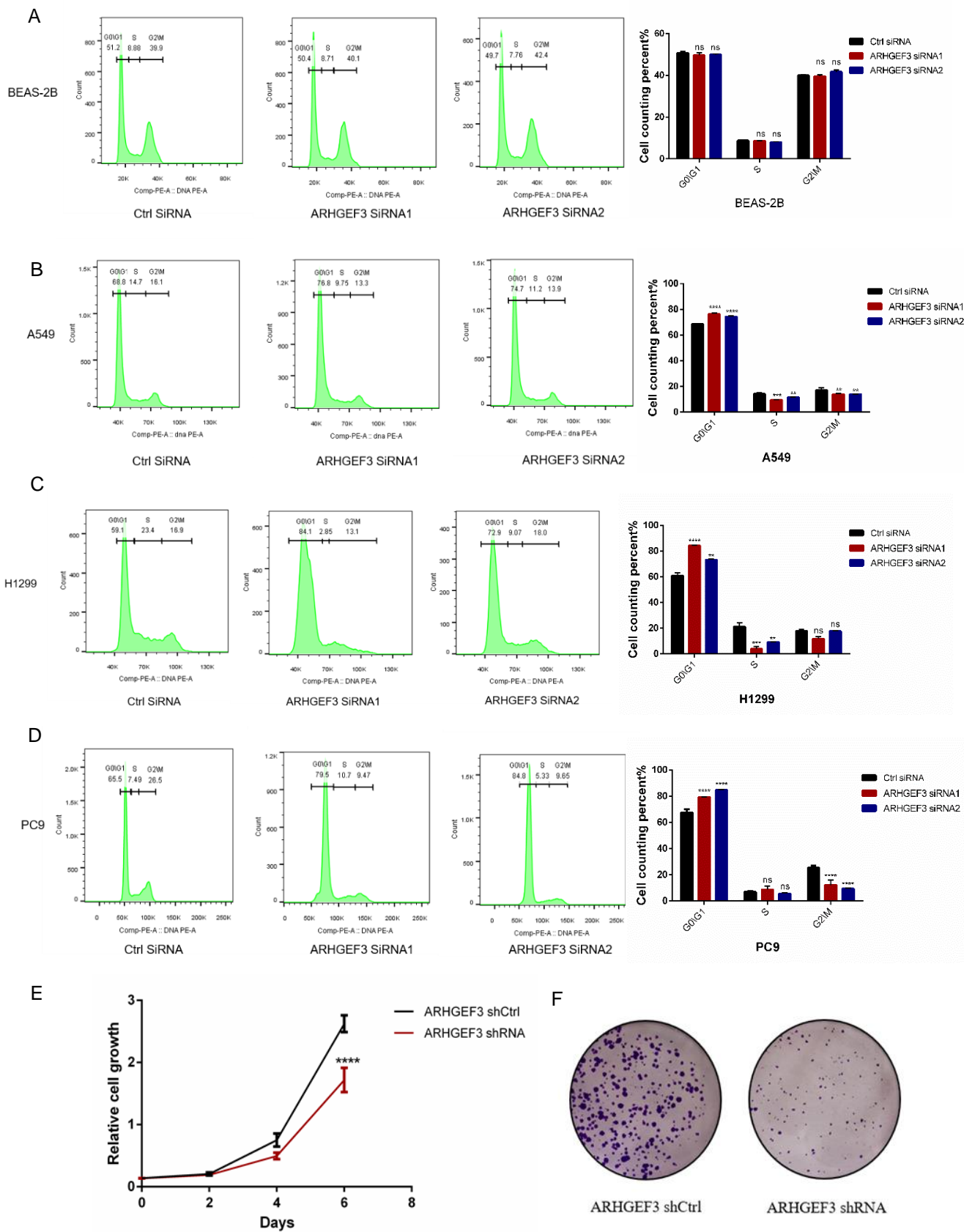

Supplement: Supplementary file 2 — Figure S1 [file 41419_2022_5297_MOESM2_ESM.pdf]

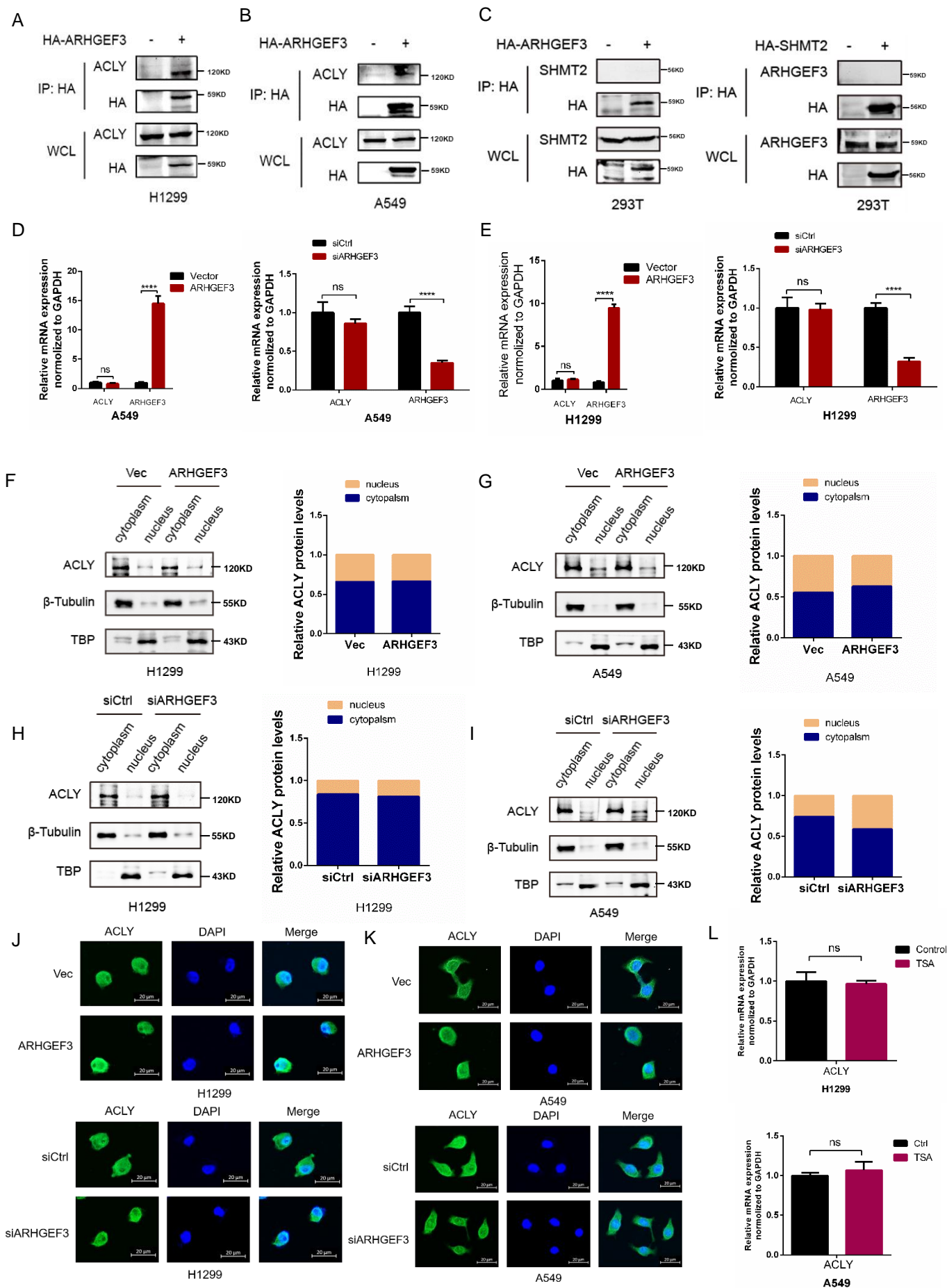

Supplement: Supplementary file 3 — Figure S2 [file 41419_2022_5297_MOESM3_ESM.pdf]

**Figure S3. Interactions of ARHGEF3 with SIRT1 and HDACs**

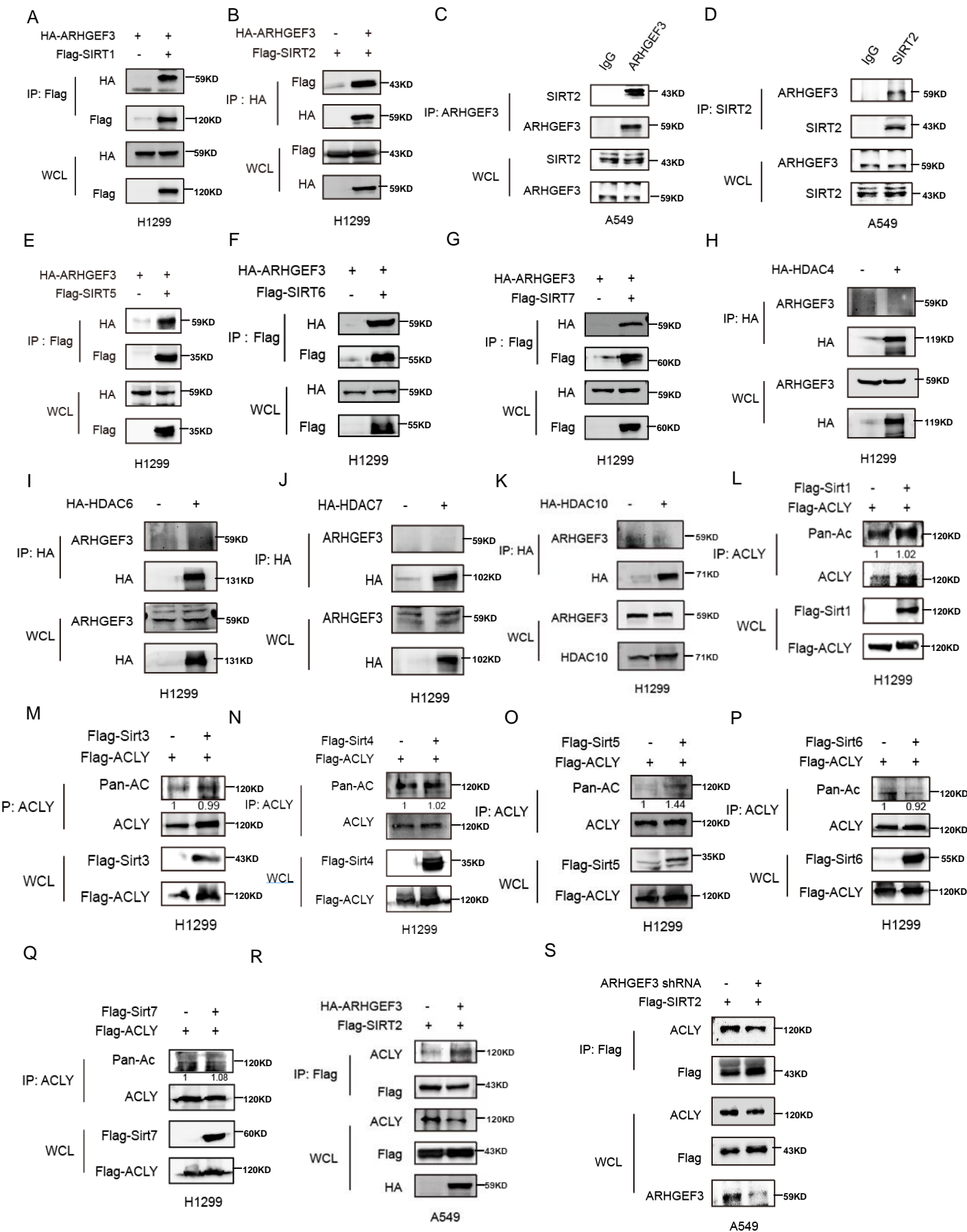

Supplement: Supplementary file 4 — Figure S3 [file 41419_2022_5297_MOESM4_ESM.pdf]

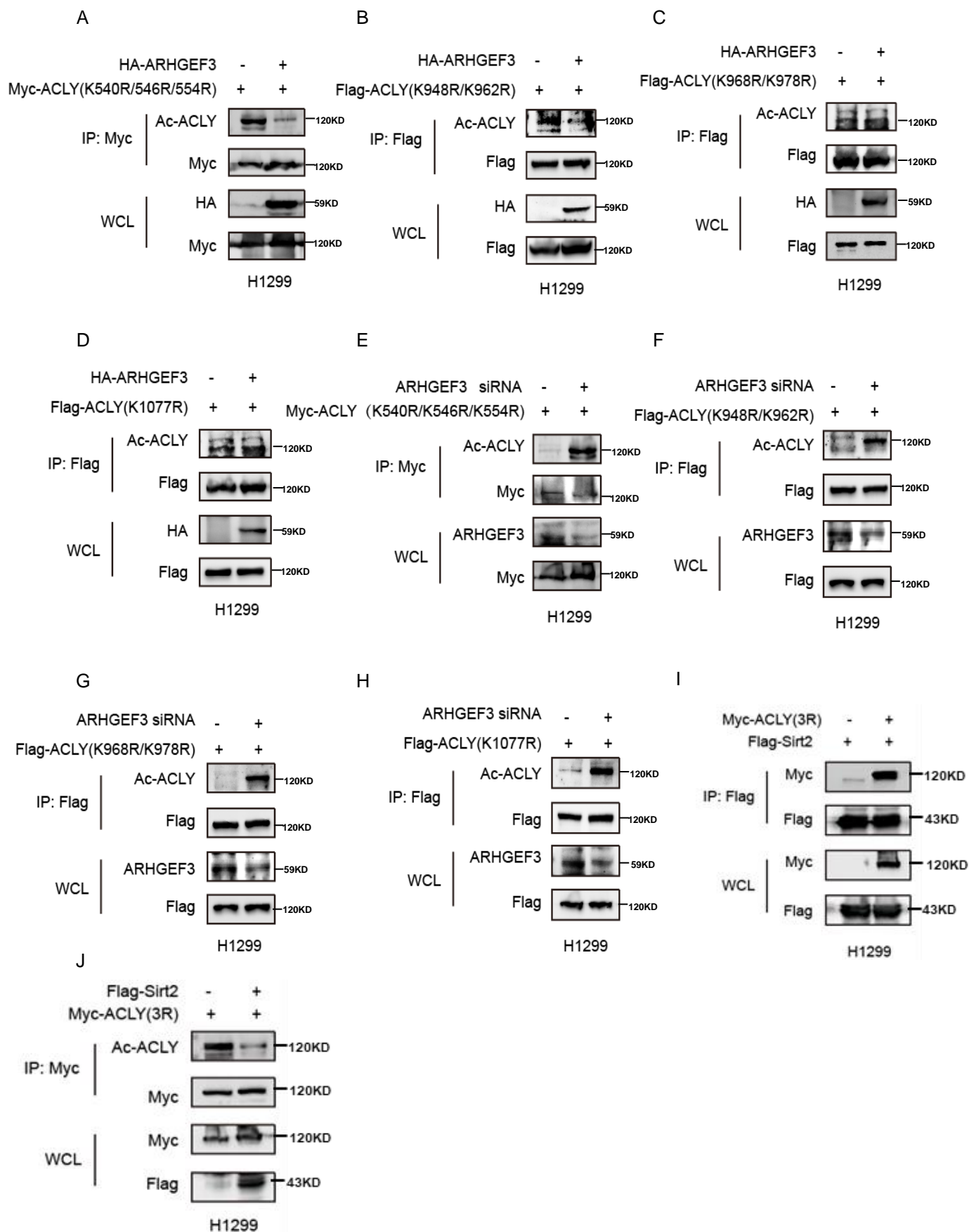

Supplement: Supplementary file 5 — Figure S4 [file 41419_2022_5297_MOESM5_ESM.pdf]

A

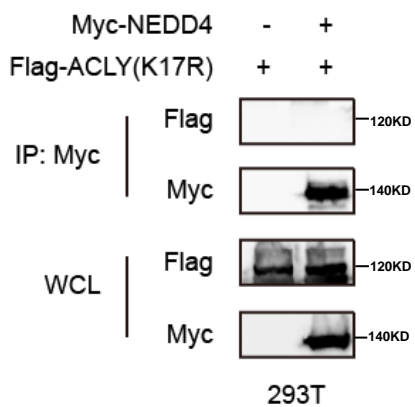

B

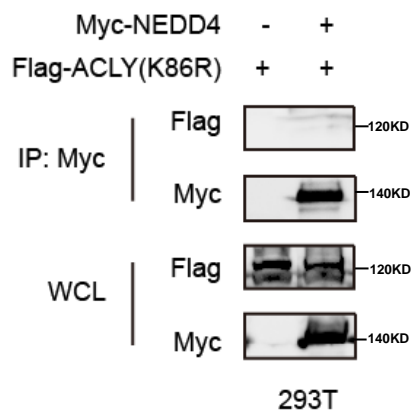

C

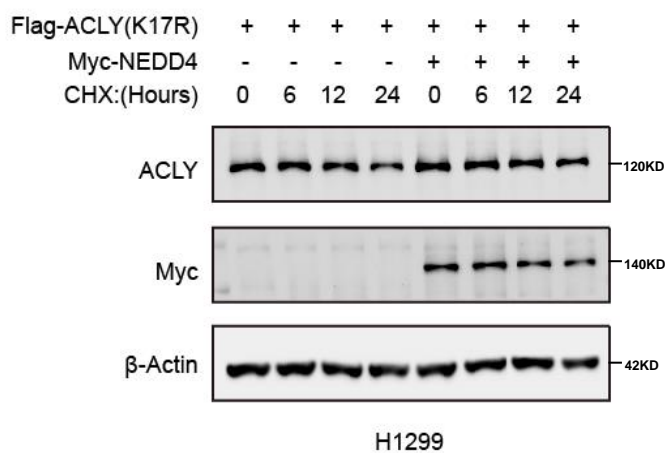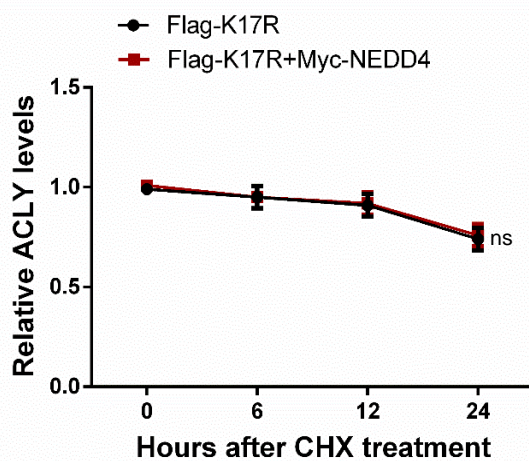

D

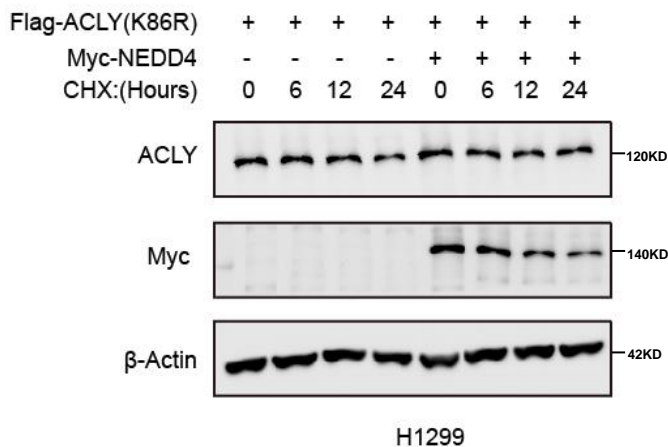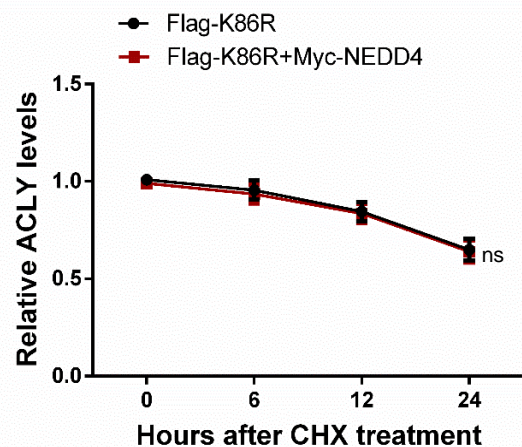

Supplement: Supplementary file 6 — Figure S5 [file 41419_2022_5297_MOESM6_ESM.pdf]
